# The relationship between physical activity, mental wellbeing and symptoms of mental health disorder in adolescents: a cohort study

**DOI:** 10.1186/s12966-019-0901-7

**Published:** 2019-12-26

**Authors:** Sarah Louise Bell, Suzanne Audrey, David Gunnell, Ashley Cooper, Rona Campbell

**Affiliations:** 1 Population Health Sciences, Bristol Medical School, University of Bristol, Bristol, BS8 2PS UK; 2 National Institute of Health Research Biomedical Research Centre at the University Hospitals Bristol NHS Foundation Trust, Bristol, UK; 3 Centre for Exercise, Nutrition and Health Sciences, School for Policy Studies, University of Bristol, Bristol, BS8 1TZ UK

**Keywords:** Physical activity, Mental wellbeing/mental health/mental illness/mental health disorder, Adolescents/young people, Cohort study

## Abstract

**Background:**

Mental illness is a worldwide public health concern. In the UK, there is a high prevalence of mental illness and poor mental wellbeing among young people. The aim of this study was to investigate whether physical activity is associated with better mental wellbeing and reduced symptoms of mental health disorder in adolescents.

**Methods:**

A cohort of 928 12–13 year olds (Year 8) from six secondary schools in England, who had participated in the AHEAD trial, ‘Activity and Healthy Eating in Adolescence’, were followed up three years later (when 15–16 years old, Year 11). At baseline, physical activity was measured using accelerometers. At follow-up, mental wellbeing was measured using the ‘Warwick Edinburgh Mental Wellbeing Scale’ (WEMWBS) and symptoms of mental health disorder using the ‘Strengths and Difficulties Questionnaire’ (SDQ). Multivariable linear regression analyses were used to investigate associations between physical activity and both mental wellbeing and symptoms of mental health disorder.

**Results:**

794 (86%) of the eligible 928 young people provided valid accelerometer data at baseline. 668 (72%) provided complete mental wellbeing data and 673 (73%) provided complete symptoms of mental health disorder data at follow-up. The multivariable analyses showed no evidence of an association between physical activity volume (counts per minute (cpm)) or intensity (Moderate to Vigorous Physical Activity (MVPA)) and mental wellbeing (WEMWBS overall score) or overall symptoms of mental health disorder (SDQ Total Difficulties Score). However, higher levels of physical activity volume at age 12–13 years were associated with lower scores on the emotional problems subscale of the SDQ at age 15–16 years.

**Conclusions:**

This cohort study found no strong evidence that physical activity is associated with better mental wellbeing or reduced symptoms of mental health disorder in adolescents. However, a protective association between physical activity and the emotional problems subscale of the SDQ was found. This suggests that physical activity has the potential to reduce symptoms of depression and anxiety in adolescents. Future cohort study designs should allow for repeated measures to fully explore the temporal nature of any relationship.

## Background

Mental illness is a worldwide public health concern [1]. It is currently the largest single cause of disability in the UK representing an estimated 28% of the total disease burden (compared to 16% each for cancer and heart disease) [2]. The World Health Organization (2013) estimates that, worldwide, 20% of adolescents in any given year may experience mental illness. In England, the most recent population survey (2017) reported 14.4% (1 in 7) of young people aged 11–16 years were identified with a mental health disorder [3]. Emotional disorders (present in 9%) were the most common type at this age followed by behavioural (conduct) disorders (6.2%) [3]. Mental health disorder has diverse and long-term negative effects on individuals, their families, and wider society [4].

Population surveys have also found increased levels of low wellbeing in young people [3]. Mental wellbeing is conceptualised as more than the absence of mental illness [5]. It has been described as encompassing hedonic (happiness, life satisfaction, and affect) and eudaimonic (positive functioning, sense of purpose, and self-acceptance) wellbeing [6–10]. Mental wellbeing is protective for a range of health outcomes [11–14] and found to be associated with higher educational outcomes in adolescence and better occupational functioning in adulthood [15–17]. Correlates of young peoples’ mental illness and mental wellbeing are reported to be largely distinct, stressing the importance of considering these concepts separately and avoiding their conflation [18].

While mental illness and mental wellbeing may be related, they are not necessarily distinct ends of a continuum [19–21]. The dual continuum model views mental illness (or mental health disorder) and mental health (or mental wellbeing) as two separate continua rather than as opposite ends of the same continuum [20]. Keyes and Lopez (2002) depicted the dual continuum model of mental illness and mental health and described four states: struggling (incomplete mental illness i.e. mental illness and high wellbeing), floundering (complete mental illness i.e. mental illness and low wellbeing), languishing (incomplete mental health i.e. no mental illness and low wellbeing), and flourishing (complete mental health i.e. no mental illness and high wellbeing). A large number of adolescents are thought to suffer from poor mental wellbeing despite being free from mental illness [4, 22]. Therefore, promoting mental wellbeing alongside preventing and treating the symptoms of mental illness, is a growing priority. The recent Green Paper (2018) focuses on schools finding low cost and low risk interventions to promote mental wellbeing and prevent symptoms of mental health disorder [23].

Although there is evidence of physical activity improving mental wellbeing [24, 25] and having the potential to prevent symptoms of mental health disorder [26, 27] in adults, the evidence of any relationship in adolescents is weaker. The studies lack measurement consistency, having defined and assessed physical activity, mental wellbeing, and symptoms of mental health disorder in a variety of ways. Furthermore, few studies have used a multi-dimensional measure of mental wellbeing or symptoms of mental health disorder (most capture only one component of mental wellbeing such as self-esteem [28] or self-efficacy [29] or a specific mental health problem such as depression [30–34]), and studies that have used an objective measure of physical activity to assess any relationship are limited [35–37].

Several reviews have attempted to analyse any association in young people [38–51]. A review of reviews by Biddle et al. (2011) [48] showed that physical activity has beneficial effects on mental health in children and adolescents. More recently there has been a significant increase in the number and quality of studies exploring any association, and when the review of reviews was updated in 2019 [49], physical activity continued to be shown to be associated with certain mental health outcomes in young people (a causal association was found with cognitive functioning, a partial association for depression, no association for self-esteem, and research focusing on the association of physical activity with anxiety was reported to be variable but generally showed small beneficial effects) [49]. A review by Rodriguez-Ayllon et al. (2019) [50] analysed the effects of physical activity interventions (randomised controlled trials and non-randomised controlled trials) on mental health outcomes of adolescents and also synthesised the observational evidence (both longitudinal and cross-sectional). Their review included studies that had at least one ‘psychological illbeing’ (i.e. depression, anxiety, stress or negative effect) and/or ‘psychological wellbeing’ (self-esteem, self-concept, self-efficacy, self-image, positive affect, optimism, happiness and satisfaction with life) outcome. They concluded that there was a small positive effect of physical activity interventions on mental health outcomes in adolescents [50].

The SDQ is a unique screening tool of symptoms indicating overall mental health disorder in young people. This composite measure identifies symptoms of emotional problems, hyperactivity, and behavioural/conduct problems and has been used in the UK series of surveys of the mental health of children and young people (1999, 2004 and 2017) [3] alongside other significant population surveys [52]. Despite this, few studies have used this measure when looking at the association between physical activity and symptoms of mental health disorder [31, 53–55]. Of the studies identified, neither of the two longitudinal studies used an objective measure of physical activity [31, 53].

The WEMWBS is a relatively new scale, also used in the series of surveys in the UK [3], designed to capture population mental wellbeing. The reviews that have included studies assessing the relationship between physical activity and various aspects of mental wellbeing [38–44; 46–50] have concluded that there is evidence of promise, but further studies are needed that use a multi-dimensional measure such as the WEMWBS.

Given the limitations of the evidence base, the aim of this study was to determine whether physical activity is associated with mental wellbeing and symptoms of mental health disorder in adolescents. This is the first study to investigate any potential relationship longitudinally using an objective measure of physical activity and valid and reliable self-report measures of both mental wellbeing, using the WEMWBS, and symptoms of mental health disorder, using the SDQ, in adolescents.

## Methods

A prospective cohort was formed based on the 928 participants from six secondary schools in the South West of England who took part in a two-year school-based exploratory randomised controlled trial (RCT) of an Activity and Healthy Eating intervention for use in ADolescence: the AHEAD trial [56]. All state secondary schools in the selected local authorities were invited to participate in the study. Schools first to express an interest (ensuring variation in size, geographical area, Ofsted rating, Free School Meal entitlement, and achievement rating) were recruited to the study. Physical activity was measured in 2008 when the participants were aged 12–13 years (Year 8); mental wellbeing and symptoms of mental health disorder was measured three years later (2011) when the participants were aged 15–16 years (Year 11). The inclusion criteria were participation in the AHEAD trial and continued attendance at a study school three years later. Data collections at baseline (2008) and follow-up (2011) were conducted by a team of researchers in the schools (classrooms or school halls) during a usual lesson (approximately 60 min). There was no evidence of promise that the AHEAD intervention improved physical activity or diet.

### Physical activity measure

The ActiGraph GT1M accelerometer (ActiGraph, LLC, Penscola, FL) was used to measure physical activity. Participants were instructed in the use of the accelerometers in school, and then asked to wear the instrument for seven days during waking hours, except for water-based activities such as bathing and swimming. Accelerometer data were downloaded using ActiLife software (Lifestyle Monitor System software Version 3.3.0) and processed using Kinesoft software (Version 3.3.62; Kinesoft, Saskatchewan, Canada) to generate outcome variables (10 s epochs were used to capture the sporadic nature of adolescent physical activity). Physical activity volume was computed as mean accelerometer counts per minute (cpm), and physical activity intensity (mean minutes per day of Moderate to Vigorous Physical Activity (MVPA)) was computed using established thresholds [57]. A valid day of measurement was defined as recording at least 480 min (8 h) of data (monitoring period from 7 am until 11 pm; periods of ≥60 min of consecutive zeros, with allowance for 2 min of interruption, was classed as nonwear time) and at least three valid days were required for inclusion in analyses.

### Mental wellbeing and symptoms of mental health disorder measures

The ‘Warwick Edinburgh Mental Wellbeing Scale’ (WEMWBS) [58], validated for use in adolescents aged 13–16 years, was used to measure mental wellbeing. The WEMWBS has 14 positively worded items with a five-point Likert scoring scale for each item (with scores ranging from 1 = none of the time to 5 = all of the time). The responses to each item were summed to give an overall WEMWBS score; a minimum score of 14 (i.e. poor mental wellbeing) and a maximum of 70 (i.e. good mental wellbeing). The higher score, indicative of better mental wellbeing, reflects more positive thoughts, feelings and behaviours. Where scores for three or less items were missing the mean value of responses for completed items for that individual was used to replace the score for missing items, enabling a total score for that individual to be computed [59]. If more than three items were missing the data for that participant were not used. The WEMWBS was selected due to it being the first multi-dimensional scale to measure population mental wellbeing in adolescents, based on established indicators. It covers most aspects of mental wellbeing including both hedonic and eudaimonic perspectives and is suitable for looking at the relationship between physical activity and mental wellbeing.

The ‘Strengths and Difficulties Questionnaire’ (SDQ) [60] was used to measure symptoms of mental health disorder. It is a behavioural screening tool used to assess social, emotional, and physical aspects of behaviour in young people [61] and has been shown to be valid and reliable for completion by 11–16 year olds [62]. The questionnaire has 25 items which comprise five subscales: (i) emotional symptoms (anxiety and depressive symptoms); (ii) conduct problems; (iii) hyperactivity/inattention; (iv) peer relationship problems; and (v) pro-social behaviour (positive behaviours such as being kind and helpful, scored in reverse of the other subscales). Response options are ‘not true, somewhat true, or certainly true’ (scored 0, 1 or 2). The SDQ ‘Total Difficulties Score’ (SDQ TDS) was generated by adding together the scores from the first four subscales and can range from 0 (low difficulties) to 40 (high difficulties). The five subscales whose scores can vary from 0 to 10 were also investigated independently. Items scores which were missing were imputed only if at least three out of five items were complete on each subscale. In this case the total subscale score for each participant was divided by the number of complete items to get the mean score and used to replace the missing item score [60, 63]. If more than two items were missing from any sub scale the data for that subscale for that participant were not used. Participants required a score for each of the SDQ subscales to be able to compute their SDQ TDS. The SDQ was selected as the SDQ TDS provides a useful indicator of the level of symptoms of mental health disorder overall. Furthermore, the subscale items may be used to indicate specific clinical disorders in adolescents: depression, anxiety, hyperactivity attention deficit disorder (ADHD) and behavioural/conduct disorder. The SDQ is a useful screening tool for identifying young people with raised scores thus potentially at risk.

### Possible confounders and mediators

The self-report behavioural questionnaires recorded a number of potential confounders and mediators of any potential relationship: age; gender; ethnicity; socioeconomic status (SES) (measured using the ‘Family Affluence Scale’ FAS II [64]); study school; number of daylight minutes (a proxy for season); baseline symptoms of mental health disorder (SDQ TDS); sleep (frequency of feeling tired when going to school in the morning); number of friends; belonging to teams and clubs; smoking; drinking alcohol; and intervention arm of the AHEAD trial [56] (the participants were randomised into two groups - the intervention arm received a physical activity and healthy eating intervention and the control arm continued with usual practice).

Confounders included were determined by the construction of a directed acyclic graph (DAG) and the availability of relevant data on study participants. Accelerometer wear time was computed from the participants accelerometer data.

Data from the self-report behavioural questionnaires were entered into a secure Access database and the accelerometer data were stored as anonymised files on a secure drive. All analyses were conducted using Stata 13 MP [65].

### Statistical analysis

The analyses assessed the association between physical activity and the measures of mental wellbeing and symptoms of mental health disorder. Multivariable linear regression analyses were used to estimate exposure effects controlling for potential confounders and mediators which were investigated by grouping them as clusters of related factors in the models: i.e. socioeconomic factors (ethnic group, SES, study school); factors that may influence the physical activity data processing (daylight minutes (when volume or MVPA exposure), minutes of wear time (when MPVA exposure only); lifestyle factors (sleep, friends, belonging to teams or clubs, drinking alcohol, smoking; measured at follow-up only); baseline symptoms of mental health disorder); and then by producing a fully adjusted model containing all of these factors. Where there was evidence of confounding or mediation (associations weakened or enhanced), further models were fitted to investigate this in more detail. Confounders adjusted for were determined by the availability of relevant data on study participants. This somewhat crude epidemiological approach was used due to there being no clear evidence of associations (and whether confounders or mediators in the relationship) in the literature. Coefficients represent the linear relationship- change in WEMWBS overall score, SDQ TDS or SDQ subscale score per unit increase in physical activity (volume or intensity). Physical activity volume was defined by accelerometer counts (a dimensionless output from the accelerometer) per minute of recording (computed as total counts recorded divided by the total minutes of valid recording over the measurement period, described as counts per minute (cpm)), whilst physical activity intensity was defined as daily minutes of Moderate to Vigorous Physical Activity (MVPA). The relationship was quantified as the change in mental wellbeing or symptoms of mental health disorder score associated with an increase of 100 cpm (e.g. an increase from 508 cpm to 608 cpm, an approximately 20% increase in physical activity volume from baseline mean of the sample); or an additional 60 min of daily MVPA. Tests for interactions were carried out to investigate whether observed associations differed by gender. There was no evidence that associations between physical activity (volume and MVPA) and either mental wellbeing or symptoms of mental health disorder (WEMWBS and SDQ) differed in males and females. The test for interaction *p*-value ranged from *p* = 0.19–0.97 so all models were based on data for males and females combined.

### Ethics approval and consent to participate

The University of Bristol Faculty of Medicine and Dentistry Ethics Committee gave full approval in 2007 for the AHEAD feasibility study and pilot trial (reference number 060702) and in 2011 for the cohort study (reference number 101119).

Consent procedures were the same in the original feasibility study and pilot trial and in the subsequent cohort study. Firstly, written consent to participation was sought from each schools’ headteacher. Secondly, letters were posted by school staff to the parents/carers of all eligible school pupils explaining the study and enclosing a reply slip to be returned if parents/carers did not want their child to participate. This ‘opt-out’ method of consent has been found to be an ethical and appropriate procedure in low-risk prevention research and avoids the low response rates and potential sampling bias when opt-in parental consent procedures are used [66, 67]. At all data collections, the young people were provided with information about the study and informed that they could ‘opt-out’ of some or all the study activities at any point and were asked to sign individual assent forms.

## Results

### Cohort study profile and baseline characteristics

**Fig. 1:**
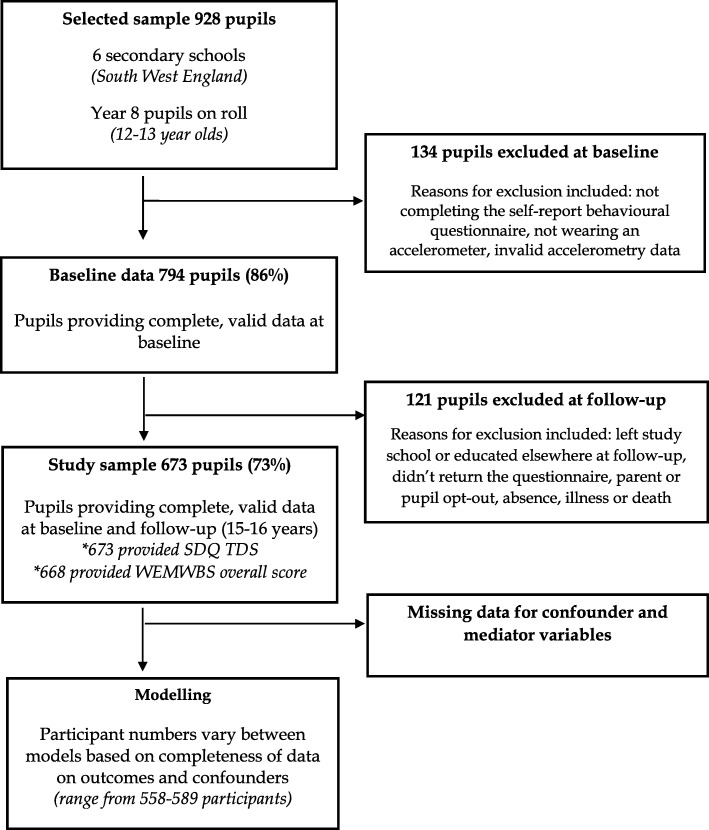
Study profile for cohort study

794 (86%) of the 928 pupils provided complete, valid baseline physical activity data and were followed-up three years later to complete mental wellbeing and symptoms of mental health disorder measures. 673 (73%) completed the SDQ and 668 (72%) completed the WEMWBS at follow-up. Those lost to follow-up were more likely to be older, male and from one particular school (due to a new headteacher using alternative educational placements for a large number of the school’s more challenging pupils). Table 1 shows the baseline characteristics of the participants included and excluded from the cohort. Figure 1 displays the study profile for the cohort study and Table 2 describes the baseline characteristics of the participants.

**Table 1:** Baseline characteristics of participants included and excluded from the cohort study

Baseline characteristics		Excluded (*n* = 242*) n (%)	Included (*n* = 673) n (%)	Difference in proportion between excluded versus included categories**
Gender	Males	149 (31.1)	330 (68.9)	X^2^ = 11.21 (*p* = 0.001)
	Females	93 (21.0)	343 (79.0)	
Ethnicity	White	217 (25.7)	629 (74.3)	X^2^ = 3.67 (*p* = 0.06)
	Other	25 (36.0)	44 (64.0)	
FAS	Low and medium (0–5)	121 (25.5)	353 (74.5)	X^2^ = 2.09 (*p* = 0.15)
	High (6–9)	87 (21.4)	320 (87.6)	
Study school	1	40 (22.0)	142 (78.0)	X^2^ = 78.04 (p < 0.001)
	2	58 (33.3)	116 (66.7)	
	3	31 (18.8)	134 (81.2)	
	4	15 (15.6)	81 (84.4)	
	5	28 (16.5)	142 (83.5)	
	6	70 (54.7)	58 (45.3)	
Physical activity***	Volume *(mean counts per minute)*	482.78 (158.12)	508.28 (169.42)	−25.5 (95% CI −58.02 to 7.02) *p* = 0.12
	MVPA *(mean daily MVPA minutes)*	51.90 (21.66)	55.59 (21.47)	−3.69 (95% CI −7.86 to 0.48) *p* = 0.08

*13 pupils were excluded but did not have baseline characteristics data to compare

**Pearson’s chi-squared tests

***Physical activity measures in excluded group n = 121 due to missing data (t-test mean difference)

**Table 2:** Baseline characteristics of participants in the cohort study

Continuous variables	All		Females		Males	
	n	Mean (SD)	n	Mean (SD)	n	Mean (SD)
Age in years	673	12.69 (0.34)	343	12.68 (0.34)	330	12.70 (0.34)
SDQ Total Difficulties Score *(SDQ TDS)*	637	12.41 (5.76)	328	12.33 (5.67)	309	12.50 (5.87)
Physical activity volume *(mean counts per minute (cpm))*	673	508.28 (169.42)	343	465.93 (153.25)	330	552.31 (174.35)
Physical activity intensity *(mean daily MVPA minutes)*	673	55.59 (21.47)	343	49.85 (19.24)	330	61.56 (22.07)
**Categorical variables**	**n**	**Category**	**All**	**Females**	**Males**	
			**n (%)**	**n (%)**	**n (%)**	
Gender	673			343 (51.0)	330 (49.0)	
Ethnicity	673	White	629 (93.0)	320 (93.3)	309 (93.6)	
		Other	44 (7.0)	23 (6.7)	21 (6.4)	
Family Affluence Scale *(SES)*	673	Low and Med (0–5)	353 (52.0)	187 (54.5)	166 (50.3)	
		High (6–9)	320 (48.0)	156 (45.5)	164 (49.7)	
Study school	673	1	142 (21.0)	79 (23.0)	63 (19.1)	
		2	116 (17.0)	64 (18.7)	52 (15.8)	
		3	134 (20.0)	62 (18.1)	72 (21.8)	
		4	81 (12.0)	41 (12.0)	40 (12.1)	
		5	142 (21.0)	59 (17.2)	83 (25.2)	
		6	58 (9.0)	38 (11.1)	20 (6.1)	
Intervention arm of the AHEAD trial	673	Intervention	339 (50.4)	184 (54.3)	155 (45.7)	
		Control	334 (49.6)	159 (47.6)	175 (52.4)	
Sleep	665	Good sleep	348 (52.0)	169 (50.0)	179 (55.0)	
		Poor sleep	317 (48.0)	172 (50.0)	145 (45.0)	
Smoking	667	Yes	115 (17.0)	71 (20.9)	44 (13.5)	
		No	552 (83.0)	269 (79.1)	283 (86.5)	
Drinking alcohol	603	Don’t drink	29 (4.8)	11 (3.5)	18 (6.2)	
		Occasional	352 (58.4)	198 (63.7)	154 (52.7)	
		Weekly	176 (29.2)	84 (27.0)	92 (31.5)	
		Frequent	46 (7.6)	18 (5.8)	28 (9.6)	
Number of friends	663	None	4 (0.6)	0 (0.0)	4 (1.2)	
		1–2	67 (10.1)	33 (9.7)	34 (10.5)	
		3–6	250 (37.7)	133 (39.1)	117 (36.2)	
		7 or more	342 (51.6)	174 (51.2)	168 (52.0)	
Belonging to teams or clubs	666	Yes	321 (48.2)	135 (39.6)	186 (57.2)	
		No	345 (51.8)	206 (60.4)	139 (42.8)	

### Physical activity at baseline

At baseline, overall physical activity volume (mean (SD) counts per minute (cpm)) was 508.3 cpm (169.42) and participants recorded 55.6 (21.5) (mean (SD)) daily minutes of MVPA). Females were less active than males with regard to both physical activity volume (mean difference 86.39 cpm (95% CI 111.2 cpm to 61.6 cpm, *p* < 0.001) and intensity (mean difference 11.7 min (95% CI − 15.41 to − 8.82), p < 0.001).

### Mental wellbeing and symptoms of mental health disorder at follow-up

There was a negative association between the WEMWBS overall score and the SDQ TDS (r = − 0.41) at follow-up. This relatively weak correlation indicates the scales are measuring different things (the WEMWBS overall score only accounts for 16% of the total variation in the SDQ TDS). At follow-up, the participants’ WEMWBS overall mean (SD) score was 48.74 (8.66) (data from a similar study (13–16 year olds) 48.8 (8.66) [58]; females (*n* = 342) 46.93 (8.90) and males (*n* = 326) 50.63 (7.99) with strong evidence of a gender difference in WEMWBS overall score (mean difference in WEMWBS overall score − 3.70 (95% CI − 4.99 to − 2.24) *p* < 0.001). Females had a lower WEMWBS overall score indicating poorer mental wellbeing than males. The participants’ SDQ TDS mean score was 12.17 (5.56) (normative data (for 11–15 years) 10.3 (5.2)) [60]; females (*n* = 343) 12.66 (5.36) and males (*n* = 330) 11.66 (5.73). Again there was evidence of a gender difference in SDQ TDS (mean difference in SDQ TDS 1.00 (95% CI 0.16 to 1.84) *p* = 0.02). Females had a higher SDQ TDS indicating higher symptoms of mental health disorder than males.

### Main findings

The univariable (adjusted for gender, age and intervention arm of the AHEAD trial) and multivariable analyses showed no evidence of an association between physical activity (volume or intensity) and mental wellbeing (WEMWBS overall score) or overall symptoms of mental health disorder (SDQ TDS) (Table 3). When the five SDQ subscales were analysed independently, an association was found between both physical activity volume and intensity and the emotional problems subscale of the SDQ (scale range 0–10). However, the association found between MVPA and the emotional problems subscale of the SDQ was slightly attenuated in the fully adjusted model (when controlling for symptoms of mental health disorder at baseline (SDQ TDS)), such that confidence intervals included the null value.

**Table 3:** Summary of unadjusted and adjusted associations between physical activity at age 12–13 years and WEMWBS and SDQ at age 15–16 years

	n*	Physical activity volume (mean cpm) Β*, 95% CI, p-value		MVPA (mean daily minutes) Β*, 95% CI, p-value	
		Unadjusted	Adjusted**	Unadjusted	Adjusted**
WEMWBS overall score	589	−0.06 (− 0.47 to 0.35) t = − 0.27 *p* = 0.79	−0.02 (− 0.44 to 0.40) t = − 0.09 *p* = 0.93	−0.54 (− 2.49 to 1.41) t = − 0.54 *p* = 0.59	0.03 (− 1.98 to 2.05) t = 0.03 *p* = 0.97
SDQ TDS	558	0.04 (− 0.24 to 0.32) t = 0.29 *p* = 0.77	− 0.16 (− 0.41 to 0.09) t = − 1.29 *p* = 0.20	−0.11 (− 1.42 to 1.21) t = − 0.16 *p* = 0.87	−0.75 (− 1.94 to 0.43) t = − 1.25 *p* = 0.21
SDQ subscales					
Emotional problems	567	−0.12 (− 0.24 to − 0.01) t = − 2.09 *p* = 0.04	−0.11 (− 0.23 to − 0.00) t = − 2.05 p = 0.04	−0.54 (− 1.08 to − 0.01) t = − 1.94 *p* = 0.05	−0.49 (− 1.02 to 0.04) t = − 1.80 *p* = 0.07
Conduct problems	559	0.06 (− 0.03 to 0.14) t = 1.29 p = 0.20	− 0.02 (− 0.10 to 0.60) t = − 0.53 *p* = 0.60	0.16 (− 0.25 to 0.58) t = 0.79 *p* = 0.43	− 0.18 (− 0.57 to 0.21) t = − 0.92 *p* = 0.36
Hyperactivity	558	0.13 (0.01 to 0.26) t = 2.18 *p* = 0.03	0.02 (− 0.09 to 0.14) t = 0.39 *p* = 0.69	0.54 (− 0.03 to 1.11) t = 1.85 *p* = 0.06	0.18 (− 0.36 to 0.72) t = 0.65 *p* = 0.52
Peer problems	567	− 0.02 (− 0.09 to 0.06) t = − 0.44 *p* = 0.66	−0.02 (− 0.09 to 0.05) t = − 0.53 p = 0.60	−0.15 (− 0.49 to 0.20) t = − 0.84 *p* = 0.40	−0.16 (− 0.51 to 0.19) t = − 0.91 p = 0.36
Prosocial behaviour	559	0.01 (− 0.08 to 0.10) t = 0.26 *p* = 0.80	0.09 (0.00 to 0.19) t = 2.00 *p* = 0.05	0.17 (− 0.27 to 0.61) t = 0.77 *p* = 0.44	0.57 (0.13 to 1.02) t = 2.53 *p* = 0.01

* Unadjusted model is adjusted for gender, age and intervention arm of the AHEAD trial

**Fully adjusted model adjusted for gender, age, intervention arm of the AHEAD trial, ethnic group, SES, study school, daylight minutes, wear time (only when MVPA the exposure), sleep, smoking, friends, belong to teams and clubs, drinking alcohol, symptoms of mental health disorder at baseline

*Coefficients represent the linear relationship between the two variables -change in WEMWBS overall score, SDQ TDS or SDQ subscale score per unit increase in physical activity (an additional 100 mean cpm or an additional 60 min of daily MVPA)

For physical activity volume, a mean increase of 100 cpm (~ 20% increase in physical activity volume) was associated with a decrease in the emotional problems subscale score of 0.12 (unadjusted model) and 0.11 (fully adjusted model). The confidence intervals (95% CI) ranged from − 0.23 to − 0.00 in the fully adjusted model implying that a potential reduction in the emotional problems subscale score of 0.23 could be achieved with an additional 100 mean cpm of physical activity (the extreme end of the effect estimate). For physical activity intensity, an additional 60 min of mean daily MVPA was associated with a decrease in the emotional problems subscale score of 0.54 (unadjusted model) and 0.49 (fully adjusted model; however confidence intervals crossed zero).

## Discussion

### Main findings

To the best of our knowledge, this is the first longitudinal study to investigate the relationship between physical activity, mental wellbeing and symptoms of mental health disorder in adolescents. We also believe this is the first study to use an objective measure of physical activity (accelerometers) and composite measures of both mental wellbeing (WEMWBS) and symptoms of mental health disorder (SDQ) validated for use with young people alongside each other, to investigate any potential associations. We found no evidence of an association between physical activity (volume or MVPA) and mental wellbeing (WEMWBS overall score) or overall symptoms of mental disorder (SDQ TDS). However, a protective association was found between physical activity volume and the emotional problems subscale of the SDQ. This finding suggests that increasing physical activity volume in adolescents may have the potential to reduce their risk of emotional problems (items on the SDQ emotional problems subscale include: worrying a lot; having fears and being easily scared; being nervous in new situations and easily losing confidence; often feeling unhappy, down-hearted or tearful; and getting a lot of headaches, stomach-aches or sickness). Those adolescents accumulating more daily MVPA minutes may have more beneficial effects. Physical activity could provide an acceptable, low risk and cost-effective intervention for young people showing symptoms of depression and anxiety.

### Strengths and limitations of the study

The study was based on a relatively large number of secondary school pupils drawn from six schools of different sizes and characteristics (e.g. rural schools, inner city schools) [56, 68]; participants are likely to be broadly representative of secondary school pupils in England. Loss to follow-up was largely due to participants no longer attending a study school and this is unlikely to have biased the patterns of associations found.

A strength of this study was its use of accelerometers, which provide an objective measure of physical activity increasing the precision of measurement. However, it is important to note that accelerometers have known limitations such as not accurately measuring cycling and not being waterproof, so do not measure physical activity from water-based activities.

This study contributes to the evolving debate concerning mental wellbeing and mental illness terminology and measurement. The findings from this study support the notion that mental wellbeing and symptoms of mental health disorder are two separate concepts, captured using different measurement scales. Using validated measures of both mental wellbeing and symptoms of mental health disorder was a strength and novel aspect of this study. This study demonstrated that physical activity is not associated with mental wellbeing or overall symptoms of mental health disorder, but has a protective relationship with regards to risk of emotional problems.

The cohort study would have benefited from repeated measures of physical activity, mental wellbeing and symptoms of mental health disorder, to fully explore the temporal nature of any relationship and take account of changing patterns of physical activity. If an effect of physical activity on mental wellbeing and symptoms of mental health disorder is short-lived, then a three-year time lag would be inappropriate to investigate any association [69].

We were able to control for a wide range of possible confounders and assess the effect of possible mediators of the relationship. However, some confounders were only measured at follow-up (sleep, number of friends, smoking, drinking alcohol, belonging to teams and clubs). The possible effects of some of the confounders may be different at baseline and follow-up (e.g. smoking and drinking behaviours would be more common at follow-up). Due to the WEMWBS not being available at the time of baseline measures, the SDQ TDS was used to control for baseline wellbeing. We were only able to adjust for confounders measured as part of the AHEAD trial. This study would have been strengthened if Body Mass Index (BMI) [70] and screen viewing behaviour [71] were measured and controlled for. The possible mechanisms underlying any association between physical activity, mental wellbeing and symptoms of mental health disorder in adolescents are complex [41]. It is possible that an integrative model that combined components of different hypotheses (biochemical e.g. release of endorphins, or psychosocial e.g. distraction, sense of mastery, social interaction) offers the most likely, full explanation [41]. Also, the effects may vary between individuals. Further research needs to consider confounding and mediating factors and exploration of potential mechanisms at the study design stage, to ensure candidate variables are measured appropriately.

### Findings in the context of existing research

Studies that have explored the relationship between physical activity and symptoms of overall mental health disorder (using the SDQ) are scarce, and of those identified, no association was reported overall [31; 53–55]. However, Sagatun et al. (2007) [53] found that for males (but not females) the number of weekly hours (self-reported weekly hours of physical activity that makes them breathless) the boys spent on physical activity per week at age 15–16 years was negatively associated with emotional problems and peer problems at age 18–19 years. Similarly, Wiles et al. (2008) [31] reported young people (aged 11–14 years) who met recommended levels for physical activity (1 h per day) had, on average, a score on the emotional problems sub-scale of the SDQ that was 0.29 units lower (− 0.29 (95%CI − 0.61 to 0.022)) at 1 year follow-up compared to those who did not undertake recommended levels of physical activity. The association found in our study between physical activity volume and the emotional problems subscale of the SDQ (− 0.11 (95% CI − 0.23 to − 0.00)) (overall but not when gender stratified) is similar to the findings of both these studies, and supports evidence from the recent physical activity and mental health in young people reviews [49, 50].

As noted at the outset, there are no longitudinal studies or RCTs that have used a multi-dimensional scale to investigate the relationship between physical activity and mental wellbeing in adolescents with which to compare our findings. Reviews have suggested evidence of promise [41, 50] but studies included only used single component measures of mental wellbeing outcomes such as self-image, satisfaction with life and happiness. In the review of reviews by Biddle et al. (2019) [49] evidence for support of a causal relationship was reported for cognitive function outcome measures, as well as academic achievement and brain structure and function. Although showing an association between physical activity and cognitive function is important to emphasis the role of physical activity in schools, this literature needs to be considered alongside further studies using a composite measure of mental wellbeing.

### Implications

There is no strong evidence of any relationship between physical activity, mental wellbeing and overall symptoms of mental health disorder in adolescents. However the findings from this study, together with that from two other studies, suggests a relationship between physical activity volume and emotional problems. Cohort studies, designed specifically to look at the relationship between physical activity and both mental wellbeing and symptoms of mental health disorder, are needed to confirm and explore any potential associations further. Measurement scales that focus on more than one aspect of mental health disorder or mental wellbeing should be used in population level school-based research, and both the SDQ and WEMWBS scales warrant further exploration. Alongside this literature, the association between physical activity and specific mental health disorders should continue to be explored. Measures that can confirm an International Statistical Classification of Diseases and Related Health Problems (ICD-*10*) or Diagnostic and Statistical Manual of Mental Disorders (*DSM*-*5*) diagnosis in young people, such as the DAWBA (Development and Well-being Assessment), should be considered. Further studies that focus on mental health disorders common in adolescents, other than anxiety and depression, are needed. An objective measure of physical activity used alongside a standardised self-report physical activity questionnaire may be the best approach to understand the context of the physical activity data (e.g. physical activity outdoors may be better for mental wellbeing and team sports may prevent a particular type of mental health disorder). It is important that the self-report measure of physical activity incorporates information on general daily living activities, such as walking to and from school or playing outdoors, and captures any relevant contextual information [70]. Future study designs should allow for repeated measures to fully explore the temporal nature of any relationship, take account of changing patterns of physical activity and look at short-term associations.

## Conclusions

This cohort study provided no strong evidence that physical activity is a protective factor for mental wellbeing or symptoms of mental health disorder in adolescents, as measured by the WEMWBS overall score and SDQ TDS. There was, however, evidence of an association between physical activity volume and the emotional problems subscale of the SDQ. This indicates that emotional problems (such as symptoms of depression and anxiety) in adolescents could be reduced by increasing their physical activity levels.

## Data Availability

The datasets during and/or analysed during the current study are available from the corresponding author on reasonable request.

## Abbreviations

AHEAD: Activity and Health Eating in ADolescence
BMI: Body Mass Index
CI: Confidence Interval
CPM: Counts Per Minute
DAWBA: Development and Well-being Assessment
DSM-5: Diagnostic and Statistical Manual of Mental Disorders, Fifth Edition) FAS (Family Affluence Scale)
ICD-10: International Statistical Classification of Diseases and Related Health Problems
MVPA: Moderate to Vigorous Physical Activity
SD: Standard Deviation
SDQ TDS: Strengths and Difficulties Questionnaire Total Difficulties Score
SDQ: Strengths and Difficulties Questionnaire
SES: Social Economic Status
WEMWBS: Warwick Edinburgh Mental Wellbeing Scale
